# Crystal structure of 4-bromo-2-[(*E*)-*N*-(2,2,6,6-tetra­methyl­piperidin-4-yl)carboximido­yl]phenol dihydrate

**DOI:** 10.1107/S2056989015007471

**Published:** 2015-04-25

**Authors:** Joel T. Mague, Shaaban K. Mohamed, Mehmet Akkurt, Antar A. Abdelhamid, Mustafa R. Albayati

**Affiliations:** a Department of Chemistry, Tulane University, New Orleans, LA 70118, USA; b Chemistry and Environmental Division, Manchester Metropolitan University, Manchester M1 5GD, England; c Chemistry Department, Faculty of Science, Minia University, 61519 El-Minia, Egypt; d Department of Physics, Faculty of Sciences, Erciyes University, 38039 Kayseri, Turkey; e Department of Chemistry, Faculty of Science, Sohag University, 82524 Sohag, Egypt; f Kirkuk University, College of Science, Department of Chemistry, Kirkuk, Iraq

**Keywords:** crystal structure, Schiff bases, piperidines, hydrogen bonding

## Abstract

In the title hydrate, C_16_H_23_BrN_2_O·2H_2_O, the organic mol­ecule features a strong intra­molecular O—H⋯N hydrogen bond. The piperidine ring, in addition, adopts a chair conformation with the exocyclic C—N bond in an equatorial orientation. The water molecules of crystallization are disordered (each over two sets of sites with half occupancy. In the crystal, they associate into corrugated (100) sheets of (H_2_O)_4_ tetra­mers linked by O—H⋯O hydrogen bonds. The organic mol­ecules, in turn, are arranged at both sides of these sheets, linked by water–piperidine O—H⋯N hydrogen bonds.

## Related literature   

For various biological applications of piperidine-containing compounds, see: Sánchez-Sancho & Herrandón (1998 ▸); Nithiya *et al.* (2011 ▸); Adger *et al.* (1996 ▸); Kozikowski *et al.* (1998 ▸); Brau *et al.* (2000 ▸).
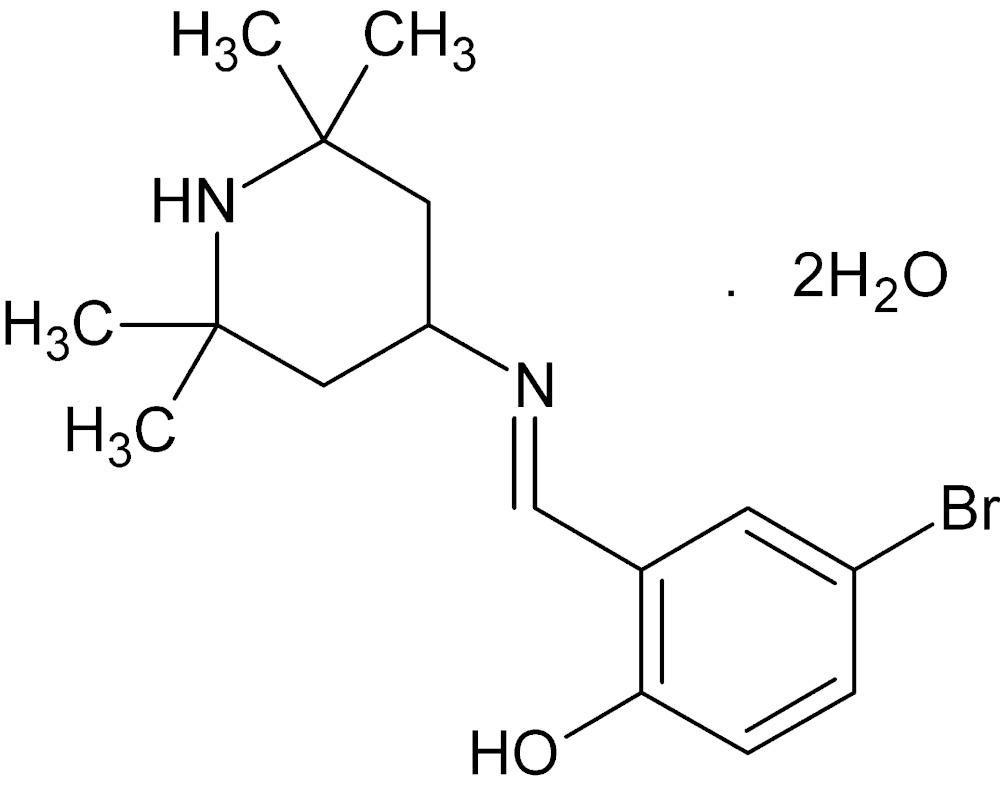


## Experimental   

### Crystal data  

C_16_H_23_BrN_2_O·2H_2_O
*M* *_r_* = 375.30
Monoclinic, 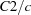

*a* = 39.6126 (7) Å
*b* = 6.0497 (1) Å
*c* = 14.8673 (3) Å
β = 98.889 (1)°
*V* = 3520.07 (11) Å^3^
*Z* = 8
Cu *K*α radiation
μ = 3.30 mm^−1^
*T* = 150 K
0.34 × 0.09 × 0.08 mm

### Data collection  

Bruker D8 VENTURE PHOTON 100 CMOS diffractometer
Absorption correction: numerical (*SADABS*; Bruker, 2014 ▸) *T*_min_ = 0.54, *T*_max_ = 0.77
12901 measured reflections
3428 independent reflections
3113 reflections with *I* > 2σ(*I*)
*R*_int_ = 0.025

### Refinement  

*R*[*F* ^2^ > 2σ(*F* ^2^)] = 0.031
*wR*(*F* ^2^) = 0.077
*S* = 1.10
3428 reflections
204 parameters
H-atom parameters constrained
Δρ_max_ = 0.56 e Å^−3^
Δρ_min_ = −0.67 e Å^−3^

### 

Data collection: *APEX2* (Bruker, 2014 ▸); cell refinement: *SAINT* (Bruker, 2014 ▸); data reduction: *SAINT*; program(s) used to solve structure: *SHELXT* (Sheldrick, 2015*a* ▸); program(s) used to refine structure: *SHELXL2014* (Sheldrick, 2015*b* ▸); molecular graphics: *DIAMOND* (Brandenburg & Putz, 2012 ▸); software used to prepare material for publication: *SHELXTL* (Sheldrick, 2008 ▸).

## Supplementary Material

Crystal structure: contains datablock(s) global, I. DOI: 10.1107/S2056989015007471/bg2553sup1.cif

Structure factors: contains datablock(s) I. DOI: 10.1107/S2056989015007471/bg2553Isup2.hkl

**Fig. 1.**
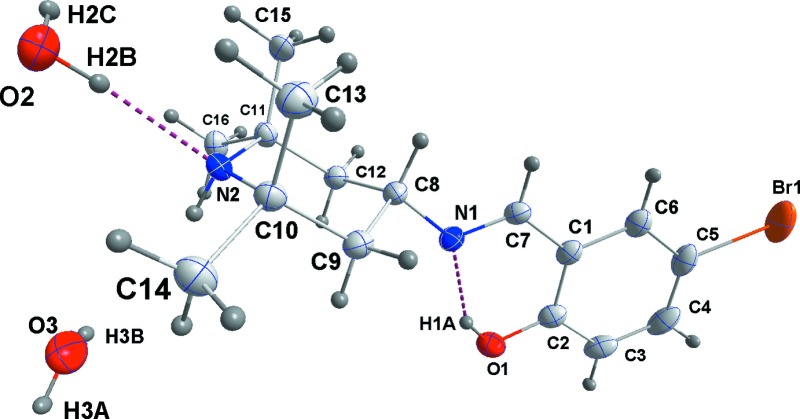
The the asymmetric unit showing the intra- and intermolecular O—H···N hydrogen bonds as dotted lines. Only one set of the disordered hydrogen atoms is shown.

**Fig. 2.**
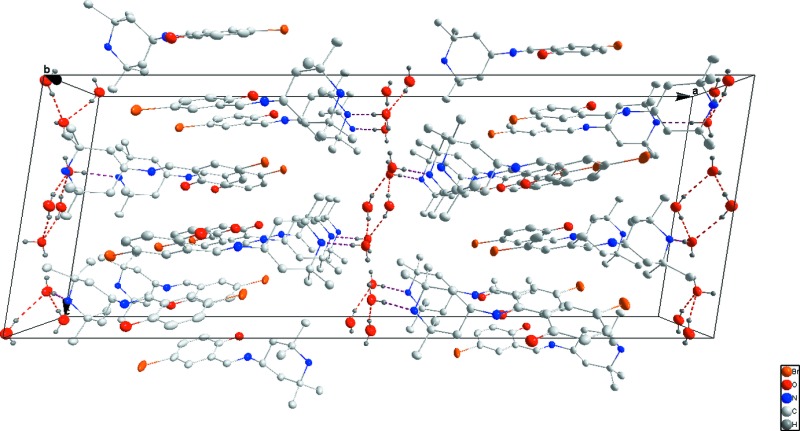
Packing viewed down the b axis with intermolecular O—H···N and O—H···O hydrogen bonds shown, respectively, as purple and red dotted lines. Only one set of the disordered hydrogen atoms is shown.

. DOI: 10.1107/S2056989015007471/bg2553fig1.tif
The the asymmetric unit showing the intra- and inter­molecular O—H⋯N hydrogen bonds as dotted lines. Only one set of the disordered hydrogen atoms is shown.

b . DOI: 10.1107/S2056989015007471/bg2553fig2.tif
Packing viewed down the *b* axis with inter­molecular O—H⋯N and O—H⋯O hydrogen bonds shown, respectively, as purple and red dotted lines. Only one set of the disordered hydrogen atoms is shown.

**Fig. 3.**
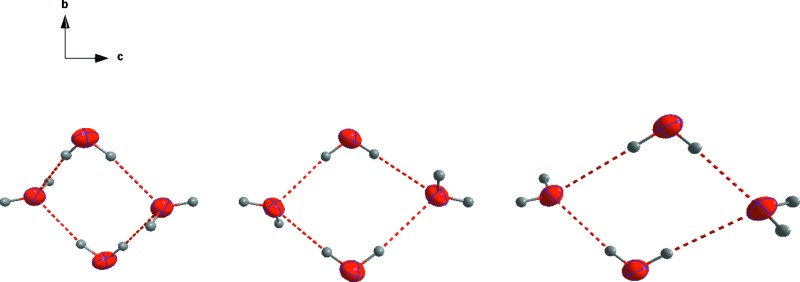
A portion of the layer of lattice water molecules. Only one set of the disordered hydrogen atoms is shown.

. DOI: 10.1107/S2056989015007471/bg2553fig3.tif
A portion of the layer of lattice water mol­ecules. Only one set of the disordered hydrogen atoms is shown.

CCDC reference: 1059897

Additional supporting information:  crystallographic information; 3D view; checkCIF report

## Figures and Tables

**Table 1:** Hydrogen-bond geometry (, )

*D*H*A*	*D*H	H*A*	*D* *A*	*D*H*A*
O1H1*A*N1	0.84	1.87	2.628(2)	149
O2H2*B*N2	0.84	2.02	2.861(2)	175
O3H3*A*O2^i^	0.84	2.24	3.059(2)	167
O3H3*B*O2^ii^	0.84	2.04	2.869(2)	168
O2*A*H2*BA*N2	0.84	2.02	2.861(2)	175
O2*A*H2*D*O3*A* ^iii^	0.84	2.04	2.869(2)	170
O3*A*H3*AA*O2*A* ^i^	0.84	2.24	3.059(2)	167

Symmetry codes: (i) 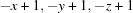; (ii) 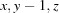; (iii) 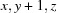.

## supplementary crystallographic information

### S1. Comment

Piperidine-bearing compounds have diverse applications in commercial and medicinal fields. The piperidine nucleus is an ubiquitous structural feature of biologically active compounds and numerous secondary metabolites, for example (S)-pipecolic acid a non-proteinogenic amino acid associated with epilepsy (Sánchez-Sancho & Herrandón, 1998; Nithiya *et al.*, 2011; Adger *et al.*, 1996). Moreover, piperidine-containing compounds were evaluated for their effect on plasma glucose level (Kozikowski *et al.*, 1998), insulin normalization and treatment of cocaine abuse (Brau *et al*., 2000). In this vein and following our strategy for synthesis of bio-active heterocyclic compounds, we report the synthesis and crystal structure of the title compound.

The conformation of the title molecule is determined in part by the strong O1—H1a···N1 hydrogen bond. The substituted piperidine ring adopts a chair conformation with puckering parameters Q = 0.503 (2) Å, θ = 12.0 (2) Å and φ = 176 (1)°. In the crystal, hydrogen bonding between the lattice water molecules generates corrugated layers approximately parallel to (100) with the piperidine nitrogen atoms (N2) hydrogen bonded to both sides (Table 1 and Fig. 2). Although the disorder in the lattice waters makes a precise description of the hydrogen bonding network in the water layer difficult (and generates apparent short H···H contacts), use of one component of the disorder indicates the presence of (H_2_O)_4_ units (Fig. 3) which hydrogen bond to the piperidine nitrogen atoms.

### S2. Experimental

A mixture of 1 mmol (156 mg) of 2,2,6,6-tetramethylpiperidin-4-amine and 1 mmol (201 mg) of 5-bromo-2-hydroxybenzaldehyde in 30 ml ethanol was heated under reflux for 5 h. The solid product was obtained on cooling, filtered off, dried under vacuum and recrystallized from ethanol to afford pale yellow columns which were suitable for X-ray diffraction. Mp. 361 K.

### S3. Refinement

H-atoms attached to carbon were placed in calculated positions (C—H = 0.95 - 0.98 Å) while those attached to nitrogen and oxygen were placed in locations derived from a difference map and their parameters adjusted to give N—H = 0.91 Å and O—H = 0.84 Å. All were included as riding contributions with isotropic displacement parameters 1.2 - 1.5 times those of the attached atoms. Each lattice water molecule is disordered over two sites with the oxygen and one hydrogen in common. Based on peak heights for the disordered H atoms, the two sites for these atoms were judged to be equally occupied.

### Crystal data

**Table:**

C_16_H_23_BrN_2_O·2H_2_O	*F*(000) = 1568
*M**_r_* = 375.30	*D*_x_ = 1.416 Mg m^−^^3^
Monoclinic, *C*2/*c*	Cu *K*α radiation, λ = 1.54178 Å
*a* = 39.6126 (7) Å	Cell parameters from 9702 reflections
*b* = 6.0497 (1) Å	θ = 6.0–72.1°
*c* = 14.8673 (3) Å	µ = 3.30 mm^−^^1^
β = 98.889 (1)°	*T* = 150 K
*V* = 3520.07 (11) Å^3^	Column, pale yellow
*Z* = 8	0.34 × 0.09 × 0.08 mm

### Data collection

**Table:**

Bruker D8 VENTURE PHOTON 100 CMOS diffractometer	3428 independent reflections
Radiation source: INCOATEC IµS micro–focus source	3113 reflections with *I* > 2σ(*I*)
Mirror monochromator	*R*_int_ = 0.025
Detector resolution: 10.4167 pixels mm^-1^	θ_max_ = 72.2°, θ_min_ = 6.0°
ω scans	*h* = −48→45
Absorption correction: numerical (*SADABS*; Bruker, 2014)	*k* = −7→6
*T*_min_ = 0.54, *T*_max_ = 0.77	*l* = −18→15
12901 measured reflections	

### Refinement

**Table:**

Refinement on *F*^2^	Primary atom site location: structure-invariant direct methods
Least-squares matrix: full	Secondary atom site location: difference Fourier map
*R*[*F*^2^ > 2σ(*F*^2^)] = 0.031	Hydrogen site location: mixed
*wR*(*F*^2^) = 0.077	H-atom parameters constrained
*S* = 1.10	*w* = 1/[σ^2^(*F*_o_^2^) + (0.0323*P*)^2^ + 4.9527*P*] where *P* = (*F*_o_^2^ + 2*F*_c_^2^)/3
3428 reflections	(Δ/σ)_max_ = 0.002
204 parameters	Δρ_max_ = 0.56 e Å^−^^3^
0 restraints	Δρ_min_ = −0.67 e Å^−^^3^

### Special details

**Table:**

Geometry. All e.s.d.'s (except the e.s.d. in the dihedral angle between two l.s. planes) are estimated using the full covariance matrix. The cell e.s.d.'s are taken into account individually in the estimation of e.s.d.'s in distances, angles and torsion angles; correlations between e.s.d.'s in cell parameters are only used when they are defined by crystal symmetry. An approximate (isotropic) treatment of cell e.s.d.'s is used for estimating e.s.d.'s involving l.s. planes.
Refinement. Refinement of *F*^2^ against ALL reflections. The weighted *R*-factor *wR* and goodness of fit *S* are based on *F*^2^, conventional *R*-factors *R* are based on *F*, with *F* set to zero for negative *F*^2^. The threshold expression of *F*^2^ > σ(*F*^2^) is used only for calculating *R*-factors(gt) *etc*. and is not relevant to the choice of reflections for refinement. *R*-factors based on *F*^2^ are statistically about twice as large as those based on *F*, and *R*- factors based on ALL data will be even larger. H-atoms attached to carbon were placed in calculated positions (C—H = 0.95 - 0.98 Å) while those attached to nitrogen and oxygen were placed in locations derived from a difference map and their parameters adjusted to give N—H = 0.91 Å and O—H = 0.84 Å. All were included as riding contributions with isotropic displacement parameters 1.2 - 1.5 times those of the attached atoms. Each lattice water molecule is disordered over two sites with the oxygen and one hydrogen in common. Based on peak heights for the disordered H atoms, the two sites for these atoms were judged to be equally occupied.

### Fractional atomic coordinates and isotropic or equivalent isotropic displacement parameters (Å^2^)

**Table:**

	*x*	*y*	*z*	*U*_iso_*/*U*_eq_	Occ. (<1)
Br1	0.82893 (2)	0.47282 (5)	0.33146 (2)	0.04137 (10)	
O1	0.69904 (4)	0.0570 (2)	0.41569 (10)	0.0300 (3)	
H1A	0.6835	0.1527	0.4080	0.056 (9)*	
N1	0.66823 (4)	0.4316 (3)	0.36510 (11)	0.0236 (3)	
N2	0.56827 (4)	0.6877 (2)	0.37146 (10)	0.0191 (3)	
H2A	0.5586	0.5638	0.3912	0.023*	
C1	0.72738 (5)	0.3664 (3)	0.35750 (12)	0.0224 (4)	
C2	0.72757 (5)	0.1514 (3)	0.39411 (12)	0.0251 (4)	
C3	0.75833 (6)	0.0340 (3)	0.40982 (13)	0.0301 (4)	
H3	0.7587	−0.1111	0.4344	0.036*	
C4	0.78818 (5)	0.1262 (4)	0.39007 (13)	0.0335 (5)	
H4	0.8089	0.0450	0.4008	0.040*	
C5	0.78768 (5)	0.3385 (4)	0.35440 (13)	0.0288 (4)	
C6	0.75773 (5)	0.4580 (3)	0.33731 (12)	0.0249 (4)	
H6	0.7577	0.6021	0.3119	0.030*	
C7	0.69657 (5)	0.5031 (3)	0.34611 (12)	0.0215 (4)	
H7	0.6977	0.6497	0.3239	0.026*	
C8	0.63930 (4)	0.5860 (3)	0.35583 (12)	0.0210 (4)	
H8	0.6465	0.7326	0.3343	0.025*	
C9	0.62799 (5)	0.6125 (3)	0.44904 (12)	0.0215 (4)	
H9A	0.6469	0.6795	0.4917	0.026*	
H9B	0.6233	0.4646	0.4728	0.026*	
C10	0.59592 (4)	0.7571 (3)	0.44580 (12)	0.0200 (4)	
C11	0.57716 (4)	0.6334 (3)	0.28012 (11)	0.0196 (3)	
C12	0.60997 (5)	0.4941 (3)	0.28757 (12)	0.0213 (4)	
H12A	0.6049	0.3418	0.3058	0.026*	
H12B	0.6173	0.4862	0.2269	0.026*	
C13	0.60480 (5)	1.0012 (3)	0.43633 (14)	0.0249 (4)	
H13A	0.6170	1.0200	0.3844	0.037*	
H13B	0.6193	1.0512	0.4920	0.037*	
H13C	0.5837	1.0889	0.4265	0.037*	
C14	0.58145 (5)	0.7278 (3)	0.53433 (12)	0.0269 (4)	
H14A	0.5617	0.8258	0.5342	0.040*	
H14B	0.5990	0.7650	0.5860	0.040*	
H14C	0.5743	0.5740	0.5397	0.040*	
C15	0.58037 (5)	0.8434 (3)	0.22423 (12)	0.0247 (4)	
H15A	0.5606	0.9394	0.2272	0.037*	
H15B	0.5811	0.8031	0.1607	0.037*	
H15C	0.6014	0.9218	0.2491	0.037*	
C16	0.54701 (5)	0.4976 (3)	0.23218 (13)	0.0252 (4)	
H16A	0.5435	0.3684	0.2694	0.038*	
H16B	0.5519	0.4485	0.1727	0.038*	
H16C	0.5263	0.5889	0.2237	0.038*	
O2	0.51008 (4)	0.9736 (2)	0.35246 (11)	0.0331 (3)	0.5
H2B	0.5278	0.8954	0.3562	0.040*	0.5
H2C	0.5038	1.0005	0.2970	0.040*	0.5
O3	0.51664 (4)	0.2904 (3)	0.49776 (11)	0.0399 (4)	0.5
H3A	0.5127	0.2158	0.5427	0.048*	0.5
H3B	0.5124	0.2081	0.4519	0.048*	0.5
O2A	0.51008 (4)	0.9736 (2)	0.35246 (11)	0.0331 (3)	0.5
H2BA	0.5278	0.8954	0.3562	0.040*	0.5
H2D	0.5105	1.0762	0.3907	0.040*	0.5
O3A	0.51664 (4)	0.2904 (3)	0.49776 (11)	0.0399 (4)	0.5
H3AA	0.5127	0.2158	0.5427	0.048*	0.5
H3C	0.5069	0.4131	0.5000	0.048*	0.5

### Atomic displacement parameters (Å^2^)

**Table:**

	*U*^11^	*U*^22^	*U*^33^	*U*^12^	*U*^13^	*U*^23^
Br1	0.01904 (12)	0.06914 (19)	0.03582 (14)	−0.00163 (10)	0.00390 (9)	−0.01717 (11)
O1	0.0346 (8)	0.0241 (7)	0.0318 (7)	0.0020 (6)	0.0068 (6)	0.0023 (6)
N1	0.0216 (8)	0.0231 (8)	0.0259 (8)	0.0039 (6)	0.0034 (6)	0.0019 (6)
N2	0.0191 (7)	0.0207 (7)	0.0184 (7)	−0.0018 (6)	0.0054 (5)	−0.0001 (6)
C1	0.0228 (9)	0.0258 (9)	0.0183 (8)	0.0036 (7)	0.0017 (6)	−0.0033 (7)
C2	0.0311 (10)	0.0250 (9)	0.0186 (8)	0.0045 (8)	0.0015 (7)	−0.0035 (7)
C3	0.0393 (11)	0.0286 (10)	0.0215 (9)	0.0126 (9)	0.0013 (8)	−0.0015 (8)
C4	0.0306 (10)	0.0446 (12)	0.0234 (10)	0.0181 (9)	−0.0020 (8)	−0.0067 (9)
C5	0.0206 (9)	0.0421 (12)	0.0227 (9)	0.0039 (8)	0.0003 (7)	−0.0109 (8)
C6	0.0228 (9)	0.0308 (10)	0.0207 (9)	0.0024 (8)	0.0023 (7)	−0.0047 (7)
C7	0.0227 (9)	0.0216 (9)	0.0202 (8)	0.0033 (7)	0.0028 (7)	0.0020 (7)
C8	0.0188 (8)	0.0189 (8)	0.0257 (9)	0.0025 (7)	0.0044 (7)	0.0041 (7)
C9	0.0226 (9)	0.0200 (8)	0.0211 (9)	0.0010 (7)	0.0014 (7)	0.0017 (7)
C10	0.0213 (8)	0.0206 (9)	0.0181 (8)	−0.0001 (7)	0.0034 (6)	0.0003 (7)
C11	0.0188 (8)	0.0224 (9)	0.0182 (8)	−0.0002 (7)	0.0046 (6)	−0.0001 (7)
C12	0.0227 (9)	0.0211 (9)	0.0206 (8)	0.0023 (7)	0.0051 (7)	0.0007 (7)
C13	0.0262 (10)	0.0188 (9)	0.0288 (10)	−0.0001 (7)	0.0018 (7)	−0.0007 (7)
C14	0.0313 (10)	0.0302 (10)	0.0201 (9)	−0.0013 (8)	0.0070 (7)	−0.0011 (8)
C15	0.0235 (9)	0.0289 (10)	0.0226 (9)	0.0030 (7)	0.0066 (7)	0.0055 (8)
C16	0.0220 (9)	0.0305 (10)	0.0232 (9)	−0.0017 (7)	0.0041 (7)	−0.0048 (7)
O2	0.0281 (7)	0.0337 (8)	0.0376 (8)	0.0058 (6)	0.0050 (6)	−0.0057 (6)
O3	0.0444 (9)	0.0318 (8)	0.0450 (9)	−0.0004 (7)	0.0113 (7)	−0.0064 (7)
O2A	0.0281 (7)	0.0337 (8)	0.0376 (8)	0.0058 (6)	0.0050 (6)	−0.0057 (6)
O3A	0.0444 (9)	0.0318 (8)	0.0450 (9)	−0.0004 (7)	0.0113 (7)	−0.0064 (7)

### Geometric parameters (Å, º)

**Table:**

Br1—C5	1.902 (2)	C10—C13	1.530 (2)
O1—C2	1.348 (2)	C11—C16	1.532 (2)
O1—H1A	0.8400	C11—C15	1.534 (2)
N1—C7	1.275 (2)	C11—C12	1.538 (2)
N1—C8	1.468 (2)	C12—H12A	0.9900
N2—C10	1.491 (2)	C12—H12B	0.9900
N2—C11	1.491 (2)	C13—H13A	0.9800
N2—H2A	0.9099	C13—H13B	0.9800
C1—C6	1.398 (3)	C13—H13C	0.9800
C1—C2	1.409 (3)	C14—H14A	0.9800
C1—C7	1.463 (2)	C14—H14B	0.9800
C2—C3	1.399 (3)	C14—H14C	0.9800
C3—C4	1.379 (3)	C15—H15A	0.9800
C3—H3	0.9500	C15—H15B	0.9800
C4—C5	1.388 (3)	C15—H15C	0.9800
C4—H4	0.9500	C16—H16A	0.9800
C5—C6	1.379 (3)	C16—H16B	0.9800
C6—H6	0.9500	C16—H16C	0.9800
C7—H7	0.9500	O2—H2B	0.8400
C8—C12	1.525 (3)	O2—H2C	0.8400
C8—C9	1.529 (2)	O3—H3A	0.8399
C8—H8	1.0000	O3—H3B	0.8400
C9—C10	1.537 (2)	O2A—H2BA	0.8400
C9—H9A	0.9900	O2A—H2D	0.8400
C9—H9B	0.9900	O3A—H3AA	0.8399
C10—C14	1.525 (2)	O3A—H3C	0.8401
			
C2—O1—H1A	107.4	C14—C10—C9	109.03 (15)
C7—N1—C8	117.69 (15)	C13—C10—C9	110.57 (15)
C10—N2—C11	119.17 (13)	N2—C11—C16	105.38 (14)
C10—N2—H2A	106.8	N2—C11—C15	111.20 (14)
C11—N2—H2A	106.4	C16—C11—C15	108.43 (15)
C6—C1—C2	119.71 (17)	N2—C11—C12	111.77 (14)
C6—C1—C7	118.73 (17)	C16—C11—C12	109.21 (15)
C2—C1—C7	121.42 (17)	C15—C11—C12	110.66 (14)
O1—C2—C3	119.07 (18)	C8—C12—C11	113.38 (14)
O1—C2—C1	121.87 (17)	C8—C12—H12A	108.9
C3—C2—C1	119.06 (19)	C11—C12—H12A	108.9
C4—C3—C2	120.81 (19)	C8—C12—H12B	108.9
C4—C3—H3	119.6	C11—C12—H12B	108.9
C2—C3—H3	119.6	H12A—C12—H12B	107.7
C3—C4—C5	119.57 (18)	C10—C13—H13A	109.5
C3—C4—H4	120.2	C10—C13—H13B	109.5
C5—C4—H4	120.2	H13A—C13—H13B	109.5
C6—C5—C4	121.09 (19)	C10—C13—H13C	109.5
C6—C5—Br1	118.76 (17)	H13A—C13—H13C	109.5
C4—C5—Br1	120.12 (15)	H13B—C13—H13C	109.5
C5—C6—C1	119.75 (19)	C10—C14—H14A	109.5
C5—C6—H6	120.1	C10—C14—H14B	109.5
C1—C6—H6	120.1	H14A—C14—H14B	109.5
N1—C7—C1	122.01 (17)	C10—C14—H14C	109.5
N1—C7—H7	119.0	H14A—C14—H14C	109.5
C1—C7—H7	119.0	H14B—C14—H14C	109.5
N1—C8—C12	109.52 (15)	C11—C15—H15A	109.5
N1—C8—C9	108.38 (14)	C11—C15—H15B	109.5
C12—C8—C9	109.97 (14)	H15A—C15—H15B	109.5
N1—C8—H8	109.6	C11—C15—H15C	109.5
C12—C8—H8	109.6	H15A—C15—H15C	109.5
C9—C8—H8	109.6	H15B—C15—H15C	109.5
C8—C9—C10	112.81 (14)	C11—C16—H16A	109.5
C8—C9—H9A	109.0	C11—C16—H16B	109.5
C10—C9—H9A	109.0	H16A—C16—H16B	109.5
C8—C9—H9B	109.0	C11—C16—H16C	109.5
C10—C9—H9B	109.0	H16A—C16—H16C	109.5
H9A—C9—H9B	107.8	H16B—C16—H16C	109.5
N2—C10—C14	106.03 (14)	H2B—O2—H2C	106.9
N2—C10—C13	110.90 (14)	H3A—O3—H3B	106.7
C14—C10—C13	108.29 (15)	H2BA—O2A—H2D	116.2
N2—C10—C9	111.84 (14)	H3AA—O3A—H3C	107.5
			
C6—C1—C2—O1	179.19 (17)	C7—N1—C8—C9	−119.20 (18)
C7—C1—C2—O1	3.7 (3)	N1—C8—C9—C10	−175.48 (14)
C6—C1—C2—C3	0.2 (3)	C12—C8—C9—C10	−55.78 (19)
C7—C1—C2—C3	−175.29 (17)	C11—N2—C10—C14	−162.17 (15)
O1—C2—C3—C4	−178.91 (17)	C11—N2—C10—C13	80.49 (19)
C1—C2—C3—C4	0.1 (3)	C11—N2—C10—C9	−43.4 (2)
C2—C3—C4—C5	0.2 (3)	C8—C9—C10—N2	49.0 (2)
C3—C4—C5—C6	−0.8 (3)	C8—C9—C10—C14	165.96 (15)
C3—C4—C5—Br1	177.42 (15)	C8—C9—C10—C13	−75.08 (19)
C4—C5—C6—C1	1.1 (3)	C10—N2—C11—C16	161.25 (15)
Br1—C5—C6—C1	−177.13 (13)	C10—N2—C11—C15	−81.47 (18)
C2—C1—C6—C5	−0.8 (3)	C10—N2—C11—C12	42.7 (2)
C7—C1—C6—C5	174.83 (16)	N1—C8—C12—C11	174.37 (14)
C8—N1—C7—C1	176.84 (16)	C9—C8—C12—C11	55.37 (19)
C6—C1—C7—N1	−178.48 (17)	N2—C11—C12—C8	−48.0 (2)
C2—C1—C7—N1	−2.9 (3)	C16—C11—C12—C8	−164.17 (15)
C7—N1—C8—C12	120.82 (18)	C15—C11—C12—C8	76.55 (18)

### Hydrogen-bond geometry (Å, º)

**Table:**

*D*—H···*A*	*D*—H	H···*A*	*D*···*A*	*D*—H···*A*
O1—H1*A*···N1	0.84	1.87	2.628 (2)	149
O2—H2*B*···N2	0.84	2.02	2.861 (2)	175
O3—H3*A*···O2^i^	0.84	2.24	3.059 (2)	167
O3—H3*B*···O2^ii^	0.84	2.04	2.869 (2)	168
O2*A*—H2*BA*···N2	0.84	2.02	2.861 (2)	175
O2*A*—H2*D*···O3*A*^iii^	0.84	2.04	2.869 (2)	170
O3*A*—H3*AA*···O2*A*^i^	0.84	2.24	3.059 (2)	167

Symmetry codes: (i) −*x*+1, −*y*+1, −*z*+1; (ii) *x*, *y*−1, *z*; (iii) *x*, *y*+1, *z*.
