# Supplementary figures and images for: Crystal structure of 4-bromo-2-[(E)-N-(2,2,6,6-tetra­methyl­piperidin-4-yl)carboximido­yl]phenol dihydrate
Source: Acta Crystallogr E Crystallogr Commun. 2015 Apr 25;71(Pt 5):o349–50. doi: 10.1107/S2056989015007471 (PMC4420100; doi:10.1107/S2056989015007471)

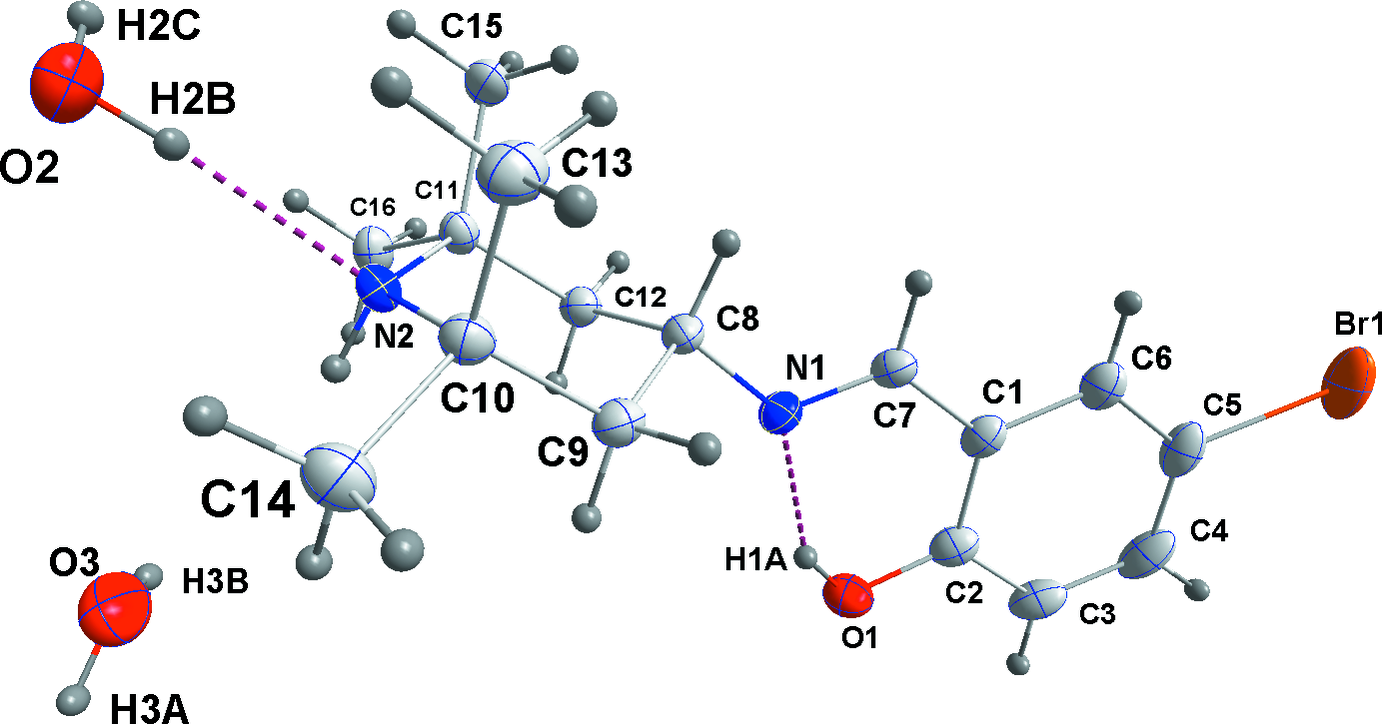

Supplement: Supplementary file 3 [file e-71-0o349-fig1.tif]

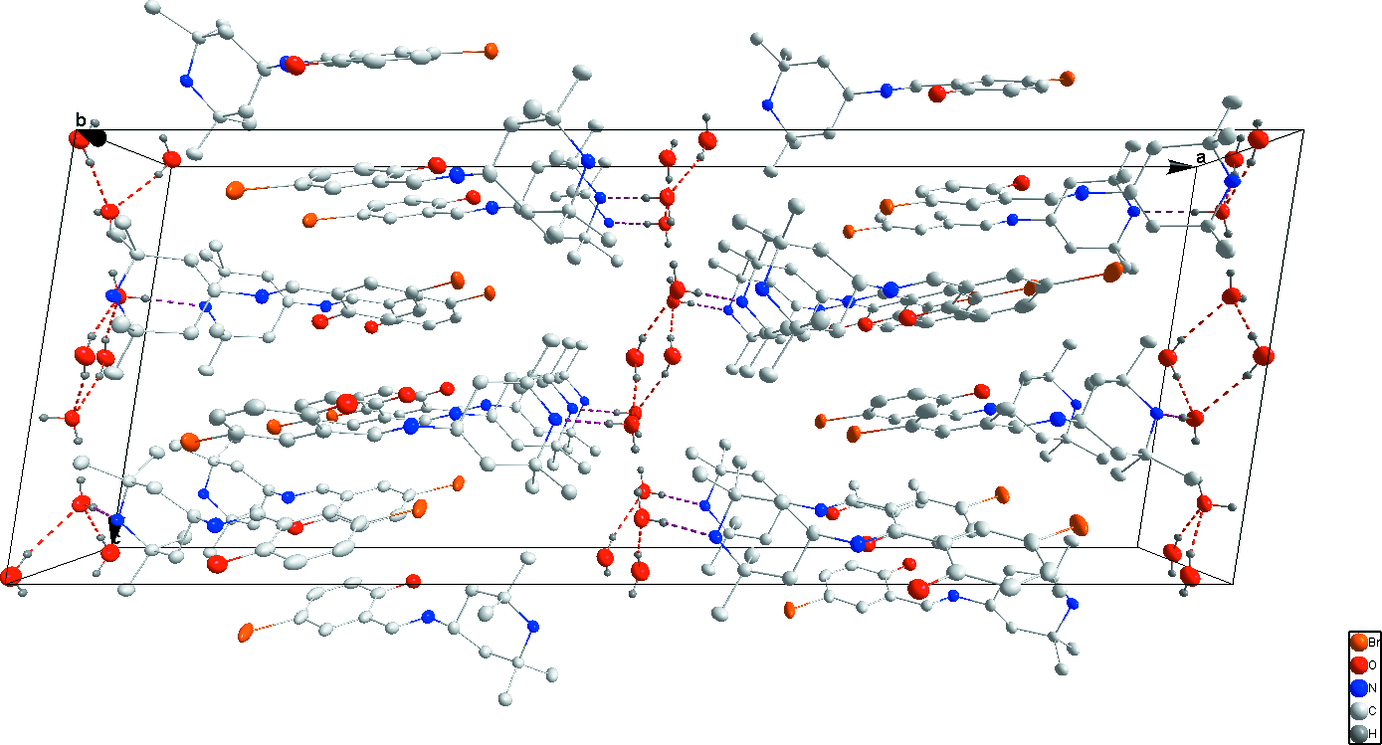

Supplement: Supplementary file 4 [file e-71-0o349-fig2.tif]

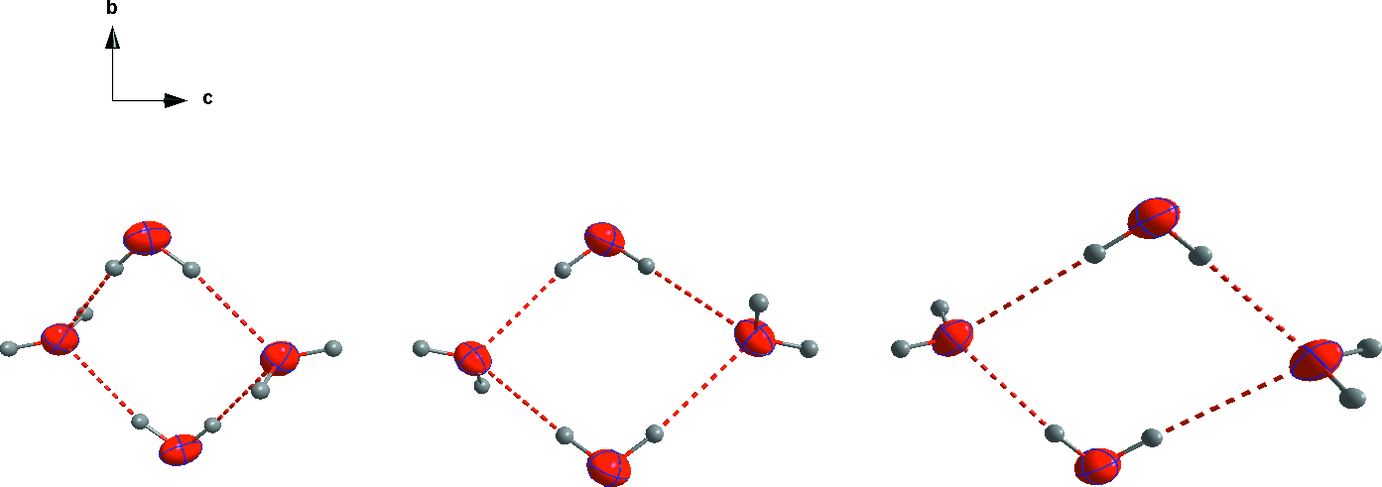

Supplement: Supplementary file 5 [file e-71-0o349-fig3.tif]
